# RISK FACTORS FOR NINE-YEAR MORTALITY OF OLDER PATIENTS WITH COGNITIVE IMPAIRMENT AT ADMISSION

**DOI:** 10.1093/geroni/igae098.2917

**Published:** 2024-12-31

**Authors:** Hiroshi Yamamoto, Kenichi Ogawa, Yasushi Hisamatstu, Tatsuya Ishitake

**Affiliations:** Yamamoto Memorial Hospital, Imari, Saga, Japan; Yamamoto Memorial Hospital, Imari, Saga, Japan; Yamamoto Memorial Hospital, Imari, Saga, Japan; Kurume University School of Medicine, Kurume University, School of Medicine, Fukuoka, Japan

## Abstract

**Background:**

Dementia can be a major cause of mortality and morbidity in geriatric patients. So, it would be essential to assess their mental state in the early. Aims: We aim to appraise the impact on mortality and hospital outcomes by revised simplified short-term memory recall test (STMT-R) and collecting clinical data simultaneous.

**Methods:**

The subjects were 612 acute inpatients ≥65 from December 2014 to September 2015. Following the collection of clinical data, survival was subsequently measured for 8-9years until December 2023. An STMT-R score of ≤4 were considered to indicate cognitive dysfunction. To explore the association between the risk factors and mortality in cognitive impairment subjects, Kaplan-Meier method and Cox’s proportional hazards regression models were used to examine mortality and survival rates.

**Results:**

The mean age of the subjects was 82.1 (±7.94), and 55.9% were female. Cognitive function was classified into three categories according to severity respectively; Incomplete Testing Group (Incomplete), Cognitive dysfunction (Abnormal) and Non-Cognitive dysfunction Group (Normal). 349 patients (57.0%) died during follow-up. Kaplan-Meier and the log-rank tests showed the negative prognostic effect of cognitive impairment, malnutrition, pneumonia and cancer-bearing state (p< 0.01). After adjustment for potential covariates the Cox regression showed that mortality hazard is increased with “Incomplete” (HR 3.526 CI 95% 2.388-5.206 p< 0.0001) and “Abnormal” (HR 1.676 1.210-2.321 p=0.0019).

**Conclusion:**

Malnutrition, pneumonia and cancer-bearing state can significantly decrease the survival rate in patients with cognitive impairment at admission. Cognitive impairment also has independently an impact on survival rate in acutely ill geriatric patients.

